# A Real-Time Diagnostic System Using a Long Short-Term Memory Model with Signal Reshaping Technology for Ship Propellers

**DOI:** 10.3390/s25175465

**Published:** 2025-09-03

**Authors:** Sheng-Chih Shen, Chih-Chieh Chao, Hsin-Jung Huang, Yi-Ting Wang, Kun-Tse Hsieh

**Affiliations:** Department of Systems and Naval Mechatronic Engineering, National Cheng Kung University, Tainan 701, Taiwan; p18141025@gs.ncku.edu.tw (C.-C.C.); p1897104@gmail.com (H.-J.H.); p16144213@gs.ncku.edu.tw (Y.-T.W.); abc890702@gmail.com (K.-T.H.)

**Keywords:** predictive maintenance, remaining useful life prediction, long short-term memory models, ship propellers

## Abstract

This study develops a ship propeller diagnostic system to address the issue of insufficient ship maintenance capacity and enhance operational efficiency. It uses the Remaining Useful Life (RUL) prediction technology to establish a sensing platform for ship propellers to capture vibration signals during ship operations. The Diagnosis and RUL Prediction Model is designed to assess bearing aging status and the RUL of the propeller. The synchronized signal reshaping technology is employed in the Diagnosis and RUL Prediction Model to process the original vibration signals as input to the model. The vibration signals obtained are used to analyze the temporal and spectral energy of propeller bearings. Exponential functions are used to generate the health index as model outputs. Model inputs and outputs are simultaneously input into a Long Short-Term Memory (LSTM) model for training, culminating as the Diagnosis and RUL Prediction Model. Compared to Recurrent Neural Network and Support Vector Regression models used in previous studies, the Diagnosis and RUL Prediction Model developed in this study achieves a Mean Squared Error (MSE) of 0.018 and a Mean Absolute Error (MAE) of 0.039, demonstrating outstanding performance in prediction results and computational efficiency. This study integrates the Diagnosis and RUL Prediction Model, bearing aging experimental data, and real-world vibration measurements to develop the diagnosis and RUL prediction system for ship propellers. Experiments with ship propellers show that when the bearing of the propeller enters the worn stage, this diagnostic system for ship propellers can accurately determine the current status of the bearing and its remaining useful life. This study offers a practical solution to insufficient ship maintenance capacity and contributes to improving the operational efficiency of ships.

## 1. Introduction

In line with the global consensus of achieving net-zero emissions from ships by 2050, shipbuilding technologies are progressively advancing toward carbon neutrality and zero-carbon vessels [1]. However, given the long lifespan of ships powered by fossil fuels, many are likely to remain in operation, making the reduction of their carbon emissions a key factor in the energy transition of the shipping industry. According to the 2023 Engine Retrofit Report of the Lloyd’s Register [2], predictive maintenance technologies can be applied to sustain ship operational efficiency and reduce carbon emissions. Predictive maintenance involves using data analysis and machine learning to anticipate mechanical failures and schedule preemptive repairs, thereby enhancing reliability and extending equipment lifespans. This approach not only improves operational efficiency but also reduces energy consumption and carbon emissions. Maintaining ships in optimal condition yields multiple benefits, including improved reliability, safety, and energy efficiency. Although mechanical failures are unavoidable, predictive maintenance technologies can be used to achieve early identification of potential faults, thereby reducing unplanned downtime, minimizing profit losses, and preventing unnecessary energy waste. By monitoring sensor signals and establishing a Diagnosis and Remaining Useful Life (RUL) Prediction Model, predictive maintenance can serve as a core technology for ensuring ship reliability. For example, when installed on a ship’s propeller to collect data through sensors, the Diagnosis and RUL Prediction Model can assess operational status, detect potential failures, and estimate its RUL.

Within such maintenance strategies, vibration analysis plays a crucial role. This non-invasive monitoring technique evaluates the condition of internal components and predicts potential failures by measuring and analyzing vibration signals generated during machine operation. Under normal conditions, machines generate specific vibration patterns, while the onset of faults changes these patterns in ways that can be directly associated with particular types of failures. Compared with other condition-monitoring methods (e.g., oil analysis), vibration analysis offers several key advantages: it provides real-time monitoring, either continuous or intermittent; supports accurate positioning; and through advanced signal processing techniques, can extract subtle failure indications from complex background noise [3].

Research on artificial intelligence (AI) applications in ships has developed rather slowly in recent years. However, scholars have begun exploring relevant topics more recently. In diagnoses and predictions, a health index is defined through a series of steps to extract fault-related information from sensor signals: (1) data collection, (2) data preprocessing, (3) feature extraction, (4) model training, and (5) model validation. For instance, Han et al. [4] proposed a data-driven model for predicting marine diesel engine failure. In their model, data were collected via sensors for subsequent analysis. Data preprocessing and feature extraction are carried out to eliminate irrelevant signals and retain features that accurately depict the target system. This step is critical for constructing the Diagnosis and RUL Prediction Model, as the effectiveness of feature extraction directly affects model reliability by reducing data redundancy and standardizing the dataset to ensure balanced contributions of different features. Therefore, fault diagnostic systems generally rely on fault features generated through signal processing [5], with common approaches including Sample Entropy (SE), Wavelet Transform, and Principal Component Analysis (PCA). The literature indicates that the PCA is among the most widely used methods for predicting machinery RUL from vibration signals [6]. PCA can improve both prediction accuracy and computational efficiency by retaining key features and minimizing redundancy. Therefore, this study integrates PCA with synchronized signal reshaping techniques to accelerate the elimination of ineffective signals and capture critical fault-related feature signals.

During model training and validation, AI reasoning models in expert systems typically require failure data at the development stage. However, in practice, obtaining failure signals is challenging, making it difficult to apply AI reasoning models in real-world contexts. Thus, recent research has increasingly focused on data-driven AI models, which can identify the inherent differences among data and initiate self-learning to differentiate between normal and failure signals without relying on expert knowledge. For instance, Zhan et al. [7] employed vibration analysis and PCA to develop a multi-class support vector machine (SVM) for diagnosing failures in cylinder heads of marine diesel engines. Xie et al. [8] used PCA as the primary step in integrating fault detection methods in marine turbines. Stefano et al. [9] applied PCA to develop a classifier for detecting and monitoring water pollutants. Yu et al. [10] developed a bidirectional recurrent neural network-based autoencoder that simultaneously encodes both normal and failure data collected from multiple sensors. They also constructed a health degradation model by establishing a health index and evaluating RUL. Sun et al. [11] proposed a Sparse Autoencoder (SAE)-based deep transfer learning network to transfer an SAE trained on historical failure data to new targets, thus enhancing RUL prediction.

The main issues in RUL prediction include (1) prediction degradation, (2) noise in sensor data, and (3) time dependence [12]. To address these issues, numerous RUL prediction techniques have been proposed [13], which can generally be classified into three types: (1) fault models based on physical mechanisms, (2) knowledge- and experience-based models, and (3) data-driven models. In the case marine machinery operating under harsh conditions, physical models often encounter significant challenges in achieving accurate RUL prediction due to the diversity and complexity of failure cases. Knowledge- and experience-based models often have a higher degree of uncertainty. In contrast, data-driven models establish correlations between the monitored states and RUL, thereby reducing reliance on expert experience [14]. Most current studies on RUL prediction use existing data for estimation [15]. In marine applications requiring real-time predictions, ship propeller components are particularly vulnerable, as they are subjected to vibration, torsion, axial, and lateral forces [16], which can lead to bearing fractures and failure. In the report from Det Norske Veritas (DNV) regarding maritime damage cases, the most common mechanical failures include damage to propeller bearings and engine bearings [17]. Such failures occur most frequently in rear bearings, primarily due to lubrication breakdown caused by substantial lateral forces and torque from the propeller. Accurate diagnosis and prediction of bearing conditions can prevent failures throughout the propeller system. Although diagnostic technologies and RUL prediction algorithms have been developed, the time-dependent nature of bearing degradation highlights a significant gap in the literature on maritime RUL prediction [18].

Since the aging of ship propeller components is time-dependent, progressive wear affects subsequent states, making the problems well suited for Recurrent Neural Networks (RNNs). For instance, Chen et al. [19] applied an RNN to predict the RUL of bearings. Traditional RNNs are suitable for handling temporal correlations in time series issues; however, in practice, when the temporal contingencies in input/output sequences span longer durations, gradient-based learning algorithms often encounter the vanishing gradient problem when learning long-term dependencies. This indicates that during the training process utilizing backpropagation through time, the gradient of the error signal decreases exponentially as the number of time steps increases. When the system maintains in the same state over multiple steps, gradients of backpropagation may vanish, preventing earlier layers from receiving sufficient effective gradient information for weight updates. As a result, conventional RNNs tend to capture short-term dependencies while encountering difficulties to learn long-distance input/output dependencies [20].

In practical application environments, such as in the health diagnosis of ship propellers, sensor signals are often affected by noise and the system itself undergoes dynamic changes. Under these conditions, traditional RNNs demonstrate limited performance, as the vanishing gradient problem reduces their robustness in learning long-distance dependencies. This suggests that even small amounts of noise or system fluctuations may significantly impede the model’s ability to learn long-term patterns, making accurate prediction difficult in complex and noisy data streams. This explains the significant decline in prediction accuracy observed in traditional RNNs when addressing prognostic issues such as RUL prediction, especially near equipment failure points [21]. Similar noise-related problems have been reported in SVR for RUL prediction. Furthermore, there are additional challenges, such as low accuracy in long-term time series prediction and excessive training time for large datasets [12].

To address long-term time series problems, this study employs an LSTM model combined with an unsupervised autoencoder architecture to design the Diagnosis and RUL Prediction Model. This model estimates the remaining life of ship bearings and completes the ship propeller diagnosis and RUL prediction system. Recent research consistently highlights the growing prominence and efficacy of LSTM models, not only in industrial predictive maintenance but also specifically for marine machinery diagnostics. For instance, recent studies by Raffak et al. [22] and Elharnaf et al. [23] showcase LSTMs’ exceptional ability to process complex time-series data from sensors, achieving high accuracy in predicting machine faults and classifying failures. Their work underscores LSTMs as a powerful tool for predictive maintenance and a benchmark for handling temporal data. This capability is particularly relevant in the maritime sector. Zhang et al. [24] further affirm the widespread use of LSTMs in analyzing vibration signals from marine engines, solidifying their status as a popular and effective method for fault detection in this domain. The unsupervised learning RUL model is capable of autonomously learning the fault features of ship propeller components, allowing it to estimate component health states and generate failure warnings without requiring extensive labeled datasets. Thus, the operational efficiency of ships is significantly enhanced, and more intelligent management technologies can be applied within the maritime sector.

This study developed a sensing platform for ship propellers to conduct accelerated aging experiments and collect vibration signals. Synchronous signal reshaping technology is adopted to eliminate ineffective signals while acquiring feature signals indicating propeller component faults. Moreover, LSTM and data-driven methods are utilized for RUL predictions, completing the ship propeller diagnosis and RUL prediction system to provide predictive maintenance for propeller components. With rapid advancements in intelligent technologies, ships are becoming increasingly smarter, safer, and more sustainable, highlighting a key direction for future maritime transport. Therefore, the diagnostic and RUL prediction system developed in this study is expected to significantly enhance vessel maintenance and drive the upgrading and transformation of the deep-sea fisheries and the shipping industries.

## 2. Materials and Methods

The proposed ship propeller diagnosis and RUL prediction system collected vibration parameters from ship propellers and integrated them via synchronized signal reshaping technology to expedite the removal of ineffective signals and capture critical fault feature signals from propeller components. Subsequently, LSTM and data-driven methods were employed to establish the Diagnosis and RUL Prediction Model to forecast the RUL, providing a reliable estimation for the health states of ship propellers.

### 2.1. Ship Propeller Sensing Platforms

**Figure 1:**
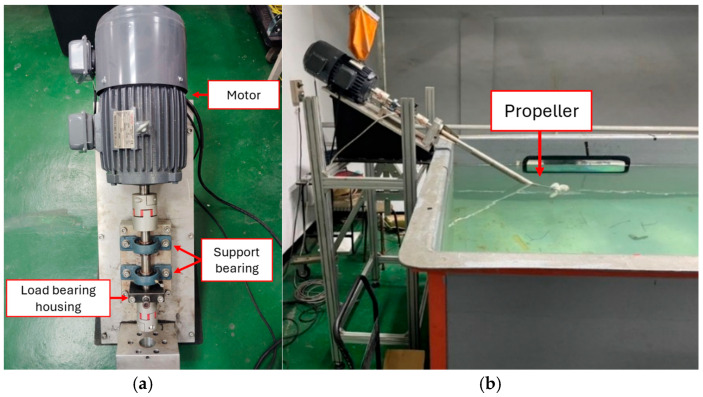
Sensing platform for ship propellers. (**a**) Top view (**b**) Side view.

The accelerated aging test for ship propeller bearings was designed to simulate and evaluate their performance and lifespan under actual operating conditions by accelerating the wear process. The sensing platform for ship propellers (Figure 1) consisted of three major modules: the bearing aging experiment module, the propeller power module, and the vibration-sensing module. The bearing aging experiment platform (Module I) comprised components such as the load-bearing housing, support bearing, propeller, and motor. Two sets of Y-bearing units were designed on the platform to ensure the shaft stability and alignment. The Y-bearing units were affixed to the base, with the rotating shaft coupled to the motor shaft. The rotating shaft passed through the load-bearing housing after going through the two sets of Y-bearing units. Aging experiments were conducted under varying loads and rotational speeds. The propeller power unit (Module II) had a power source of 2 HP 6P, fully enclosed horizontal motor with a maximum rotational speed of 1144 rpm, coupled with a 12.4 cm diameter propeller with three blades. The stern shaft was a key component that connected the propeller to the power source, and it transmitted power to rotate the propeller in water and generate thrust. The vibration-sensing module (Module III) had an IMI 607A01 accelerometer since the vibration acceleration of ship propeller under varying navigation conditions ranged from 0.1 g to 30 g. This sensor provided a measurement range of ±50 g and a sensitivity of 100 mV/g and the ability to detect vibration as small as 350 µg. The signal acquisition card was configured to accommodate the vibration frequency of the ship’s propeller shaft. The sampling rate was set at 10 kHz to ensure that high-frequency vibration features were captured. Therefore, this study employed the NI 9234 signal acquisition card, which has a wide frequency response range of up to 51.2 kHz that can cover the frequency range of ship propeller vibrations.

As the propeller bearings rotated, vibration signals were captured by the accelerometer through the signal acquisition card and signal processing equipment. Synchronous signal reshaping technology was applied to decomposed long-time series signals into multiple segment combinations. This process was equivalent to transforming the raw data into a two-dimensional matrix, enabling the LSTM model to be trained with more continuous data information, thus reducing data analysis and fault diagnosis model training time.

### 2.2. Diagnosis and RUL Prediction Model

**Figure 2:**
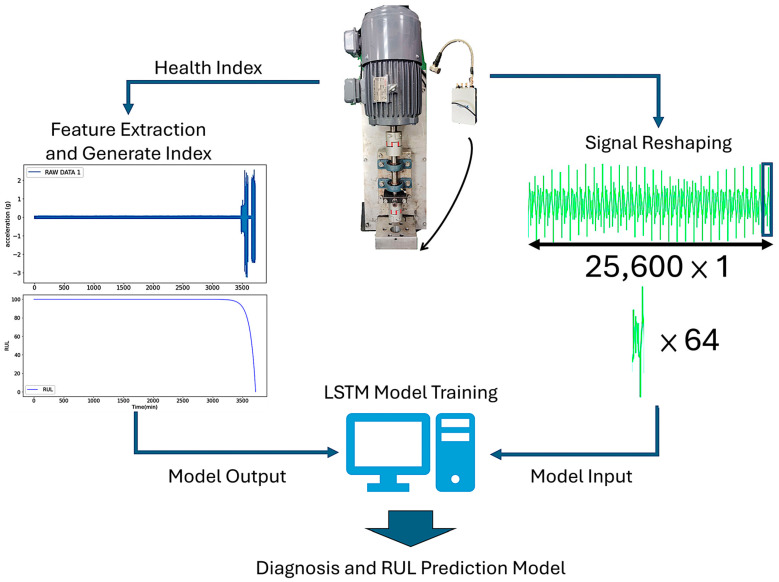
Diagnosis and RUL Prediction Model construction process.

The construction process of the Diagnosis and RUL Prediction Model is illustrated in Figure 2. Captured vibration signals underwent time-series and spectrum processing and analysis to derive a health index, which served as the output model. At the same time, reshaping the vibration signals also yielded feature signals of component failures, which served as the input model. For long-term time series signals, this study integrated LSTM with an unsupervised autoencoder architecture. The input–output model was trained using the LSTM. Based on the performance evaluation results, the model demonstrating the best performance was the Diagnosis and RUL Prediction Model developed in this study.

#### 2.2.1. Health Index: RUL Value

The vibration magnitude of machinery during operation is related to the degree of component damage. The degree and type of component damage can be estimated through measuring and analyzing vibration signals during equipment operation. Assuming that when the component’s vibration signal is *N* in length, the time series signal x = [*x*1, *x*2…, *xN*], its root mean square value serves as a comprehensive indicator. However, relying solely on statistical metrics only allows for identification of whether damage exists. To improve recognition accuracy, this study introduced frequency domain feature extraction, which highlights periodicity and frequency features within the time series signal data and distinguishes component damage types. Since spectral features vary under different rotational speeds, this study adopted the frequency band segmentation method to reduce the resolution of specific bands from the original spectrum. Lengths of the particular bands were determined by multiplying the bearing’s rotational frequency (ω) by a fixed harmonic multiple (Nh). This method allowed for varying rotational speeds to correspond to different frequency band lengths. Next, the reframing of the original spectrum could yield normalized frequency domain features that were not subject to the effects of rotational speed. The frequency band segmentation is expressed in Equation (1) as follows:

(1) $$F\left(m\right)=\int_{f_{\omega }\cdot \left(m-1\right)}^{f_{\omega }\cdot m}f\left(s\right)ds,\;\;\;\;\;m\in \left\{1,2,3,\ldots \ldots N\right\}$$

where *N* is the total number of frequency band segments. $f_{\omega }$ is the reduced frequency resolution $\left(f_{\omega }=\omega \cdot N_{h}∕N\right)$ after frequency band segmentation. f(s) is the corresponding amplitude values.

Once the feature signals were extracted, they underwent feature normalization and are integrated with the feature data. Min–Max Normalization was adopted to scale the data within the designated range [0, 1]. The Min–Max Normalization method is outlined in Equation (2).

(2) $$x_{norm}=\frac{x-min(x)}{\max\left(x\right)-min(x)}$$

Raw vibration signals may contain substantial noise, particularly interference from AC signals. Therefore, this study used a notch filter to eliminate the 60 Hz AC power frequency and its frequency multiplication. Next, Envelope Spectrum Analysis was employed to analyze the signals, as this technique is specifically designed for diagnosing local faults in mechanical components such as roller bearings and gears [8,21]. Rolling faults often generate high-frequency impacts that may induce structural resonance. Diagnostic information is not derived from the resonance frequency but rather from the frequency of repeated impacts, such as the frequency at which rolling elements pass the fault point. The original vibration signal spectrum is often masked by high-frequency resonance or background noise, making direct analysis insufficient for detecting critical fault repetition frequencies. Hence, the Envelope Spectrum Analysis was used in this study to extract the envelope of the bandpass-filtered signal through amplitude demodulation and performing spectral analysis. This method can effectively reveal weak periodic fault frequencies, allowing for stable detection even under minor random fluctuations. Another feature is the RMS value. Under normal conditions, the RMS value remains relatively stable, whereas significant and erratic increases in the RMS value indicate the presence of anomalies or faults within the equipment. This study employed the feature signal chaining method for data fusion, combining signals from multiple sources to enhance model performance and reduce the risk of overfitting. Finally, after merging the feature data, the Principal Component Analysis (PCA) was applied for signal dimensionality reduction, decreasing the number of features while retaining the number of the most representative signals. After analyzing the signal features, a health index of RUL value was established. It provides an intuitive method for monitoring the equipment’s health states and assisting in RUL prediction. As the bearings begin to deteriorate, the health index value starts to decline. This can be described as an exponential model representing the system’s RUL, as shown in Equation (3).

(3) $$RUL=-a\cdot \exp\left(\left|b\right|\cdot t\right)$$

In Equation (3), RUL represents the remaining useful life, t is time, and a and b are model parameters, with a denoting the initial lifespan and b denoting the rate of degradation.

#### 2.2.2. Diagnosis and RUL Prediction Model Training

**Figure 3:**
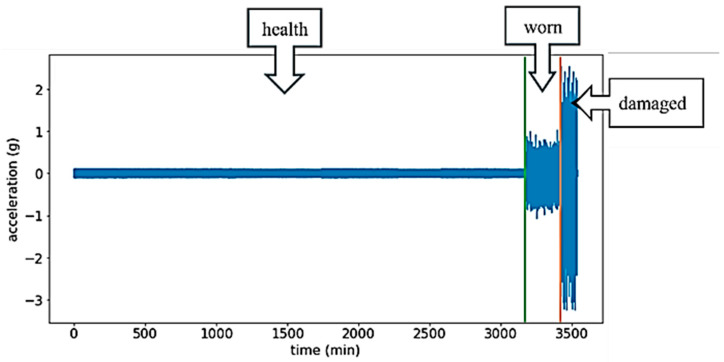
Signal graphs of rotate bearings in healthy, worn, and damaged stage.

For training the diagnosis and prediction model, continuous vibration signals of bearings in three states, namely healthy, worn, and damaged, obtained from the accelerated aging experiments, were utilized in this study, as shown in Figure 3. Data collection spans 3540 min at a sampling rate of 25,600 Hz, resulting in 5.43 billion sample points. The accelerated aging experiment platform was developed based on the architecture of the PRONOSTIA bearing test platform, utilizing the 6902 Z bearing with an experimental motor speed of 800 rpm.

To effectively accelerate the computation speed of the model, this study employed the synchronous signal reshaping technology to decompose long-time series signals into multiple segment combinations, effectively transforming the original data into a two-dimensional matrix. It is assumed that there is an original one-dimensional time series signal *x*, with a length of L:

(4) $$x=\left[x_{1},x_{2},\ldots x_{L}\right]$$

The goal is to convert this one-dimensional vector x into a two-dimensional matrix X, whose dimensions are N × M:

(5) $$X=\left[\begin{array}{c} X_{1}\\ X_{2}\\ \vdots \\ X_{N} \end{array}\right]=\left[\begin{array}{c} \begin{array}{c} x_{1}\\ x_{M+1} \end{array}\\ \begin{array}{c} \vdots \\ x_{\left(N-1\right)M+1} \end{array} \end{array}\begin{array}{c} \begin{array}{c} x_{2}\\ x_{M+2} \end{array}\\ \begin{array}{c} \vdots \\ x_{\left(N-1\right)M+2} \end{array} \end{array}\begin{array}{c} \begin{array}{c} \cdots \\ \cdots \end{array}\\ \begin{array}{c} \ddots \\ \ldots \end{array} \end{array}\begin{array}{c} \begin{array}{c} x_{M}\\ x_{2M} \end{array}\\ \begin{array}{c} \vdots \\ x_{NM} \end{array} \end{array}\right]$$

Taking vibration signals from the propeller bearing, for example, each second of vibration data, 25,600 points in length, is divided into 400 segments, each containing 64 data points, representing signal behavior over a very short time interval. When processing these segments, the LSTM model can focus on local features over short durations, such as shock signals or periodic variations. These critical features generally exhibit higher energy or clearer structures than the random noise. In contrast, noise within long-time series is typically random and irregular so that its impact within each small segment may be diluted or disregarded by the model. Moreover, when the original signal is transformed into a 400×64 matrix, it provides the model with 400 independent observation samples. During training, the LSTM model learns common patterns from these 400 samples. If a particular segment is adversely affected by random noise, it can be considered an “outlier”. However, the model can learn more generalized and stable fault patterns from the remaining 399 samples, thus effectively mitigating the impact of any single noise segment.

In the design of signal reshaping, this method divides and reorganizes time-domain signals while fully preserving the relative phase information within segments. This can enable the model to accurately capture the details and features of local time-domain waveforms. However, with long sequences decomposed into multiple discrete segments, the absolute phase relationships between segments are disrupted. This may shift the model’s learning focus from lengthy global temporal dependencies toward more diagnostically significant local feature capture and tracking of long-term trends. Nevertheless, this method does not impair the learning capability of frequency-domain features. When processing these time-domain segments, the LSTM model can still summarize frequency-related patterns and track low-frequency, long-duration trend changes, such as a gradual increase in amplitude caused by faults.

It is worth noting that the selection of this parameter combination for reshaping was an empirical combination of parameters that yielded the best results after systematic attempts in this study. Moreover, various decomposition configurations, such as 800 × 32, 200 × 128, 100 × 256, were explored. If segments are divided too finely (e.g., 800 × 32), although the number of training samples increases, each short segment may be insufficient to cover a complete fault cycles or capture key features, leading to reduced data representativeness. Conversely, if segments are divided too coarsely (e.g., 100 × 256), while a single segment contains more information, the reduced number of training samples may limit the LSTM model’s capacity to learn trends in fault evolution.

Next, LSTM training was conducted, using the reshaped vibration signal as the input of LSTM and the health index as the output of the model. The architecture and parameters of the LSTM are outlined as follows: utilize PyTorch (version 1.13.1) as the development framework, configure the model with two LSTM layers, incorporate a 10% dropout layer, and finally output the prediction results through three dense layers. The numbers of neurons in these layers were set to 256, 16, and 1, respectively, with the epochs set at 100, the batch size at 5, and the learning rate at 0.005. The dataset was divided into a training set and a validation set in a 7:3 ratio.

RNN, SVR, and Random Forest were selected as baseline models for comparison to effectively highlight the advantages of the LSTM-based approach proposed in this study. The primary objective was to validate the feasibility and effectiveness of integrating signal reshaping with the LSTM model for the RUL prediction of ship propellers. To highlight the core contributions of this study, the proposed algorithm was compared with the representative traditional machine learning and baseline time series models to provide a clear demonstration.

**Figure 4:**
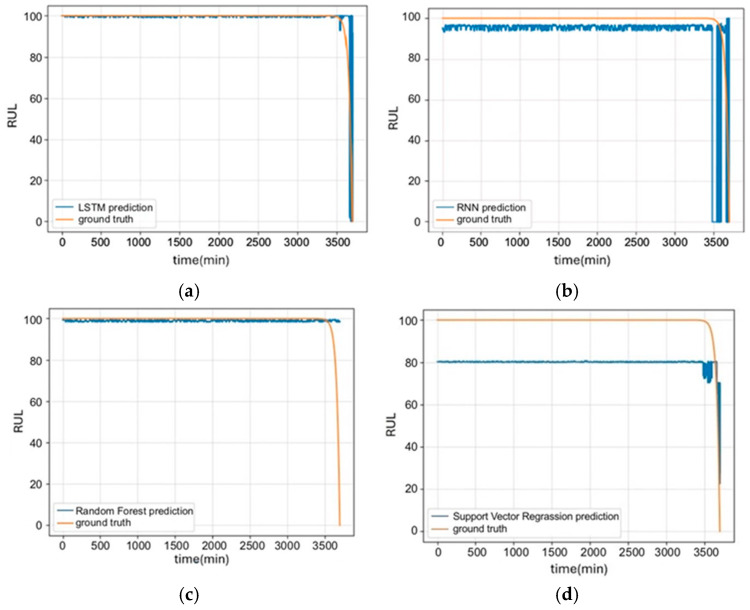
(**a**) Prediction graphs of LSTM, (**b**) RNN, (**c**) Random Forest, and (**d**) SVR models.

The trained LSTM model was used to predict the RUL of the equipment. A comparison of LSTM prediction with other models against actual measured signals is presented in Figure 4. Comparison results revealed that LSTM almost aligned with actual values, whereas RNN predictions exhibited significant deviation, particularly near fault points where prediction accuracy significantly declines. Figure 4c compares the LSTM and Random Forest, indicating that the prediction results using Random Forest significantly deviated from actual values, particularly during the fault stage. While Random Forest had certain advantages in handling nonlinear problems, its performance in processing time series data was inferior to that of LSTM. Figure 4d compares LSTM and SVR, indicating that SVR prediction results also exhibited considerable errors, suggesting that its predictive capability in time series data was inferior to that of LSTM. Among the comparisons, LSTM’s prediction results aligned most with the actual values. This finding indicates that this method not only enhanced prediction accuracy but also aided in the timely detection of faulty equipment components, allowing for preventive measures to enhance the operation reliability and extend the RUL of equipment.

This study used Mean Squared Error (MSE) and Mean Absolute Error (MAE) to assess the loss functions and evaluation metrics of LSTM performance; these served as indicators to evaluate the prediction capability and reliability of LSTM. Table 1 presents a comparison of specific performance metrics obtained from predictions using different models. Among several common machine learning models, LSTM’s average MSE value (0.018) was significantly lower than that of other models, suggesting that LSTM had the smallest SME from actual values and the highest predictive accuracy, particularly in capturing subtle changes during the aging process. Compared to the MAE, LSTM also performed best, with a value of 0.039. This result confirmed that LSTM can provide stable and accurate prediction results. Therefore, LSTM was selected as the Diagnosis and RUL Prediction Module for forecasting and diagnosing the health indexes of ship propeller shafts.

As shown in Figure 4b, RNN prediction results exhibited considerable deviation from actual values, especially when approaching fault points, where the accuracy of predictions significantly declines. This likely indicates that when employing gradient-based learning algorithms, the gradient of the error signal diminishes exponentially during backpropagation. As a result, the network weights at earlier layers, distant from the output layer, may be almost impossible to update. Consequently, the model becomes sensitive to noise or measurement errors, leading it to predict an RUL of 0 in only less than 3500 min.

Figure 4c shows that Random Forests excel in handling large datasets, high-dimensional feature spaces, and non-linear relationships, with robustness against missing data and noise. However, they cannot capture long-term dependencies in time series. In scenarios requiring the understanding of complex patterns as data evolves over time, this limitation reduces its effectiveness compared to models like RNN, which are explicitly designed for sequential data.

As shown in Figure 4d, the RUL values predicted by the SVR model exhibited a bias from the outset. As a regression model, SVR identified an optimal hyperplane that fit the data points while allowing for a certain degree of tolerance. If the input signals had slight fluctuations or noise during the healthy phase, SVR took these fluctuations into consideration. As a result, predicted values did not accurately represent 100 during the healthy phase, thus affecting the evaluation indicators.

After training the Diagnosis and RUL Prediction Model, a prediction model suitable for actual operating conditions was established. This model was applied in practical tests on ship propeller bearings. First, actual vibration signals were captured and reshaped to serve as inputs, which were fed into the trained Diagnosis and RUL Prediction Model, resulting in the health index curves. An exponential model (Equation (3)) was used to fit these curves to predict the RUL until the damage threshold was reached, completing the construction of the ship propeller diagnosis and RUL prediction system.

## 3. Results

### 3.1. Bearing Abnormal Frequency Experiment

**Figure 5:**
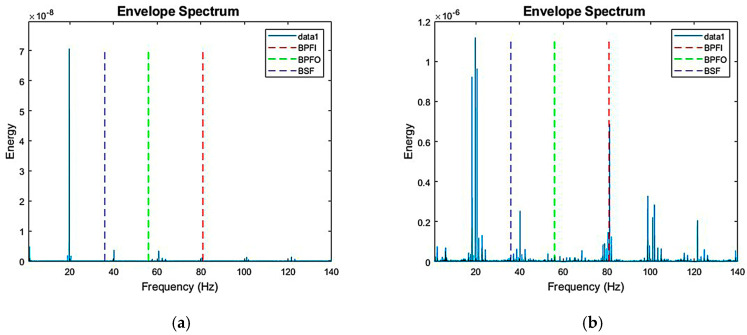
(**a**) Spectrum plot of healthy bearings; (**b**) spectrum plot of damaged bearings.

Abnormal bearing frequencies are tested under conditions such as outer race wear, inner race cracking, and ball surface degradation. By comparing the frequency spectrum of healthy bearings (Figure 5a) with that of damaged bearings (Figure 5b), the spectral features of each condition can be analyzed, facilitating the identification of vibration features associated with different bearing states. Figure 5a illustrates the spectrum under normal operating conditions: the components and energy distribution of the feature frequencies are relatively stable, and there are no significant abnormal peaks. This indicates smooth operations without prominent damage. Figure 5b shows wear on the outer race and bearing balls, with significant cracks in the inner race. The spectrum reveals higher-order harmonics at the Ball Pass Frequency Inner Race (BPFI), ranging from 79 to 83 Hz, confirming the influence of inner race damage on the feature frequency. Additionally, wear on the outer race and bearing balls is reflected in the spectrum plot at 64–68 Hz and 43–47 Hz, respectively. Although these are not as obvious as the cracks in the inner race, corresponding changes in frequency components can still be observed.

The spectrum plot of damaged bearings reveals abnormal peaks at non-feature frequencies, primarily due to the following reasons: (1) structural resonance, which may induce additional vibrations during operation, and (2) higher-order harmonics, caused by cracks or defects in the rolling elements, inner race, or outer race. Such damage may generate nonlinear effects that lead to higher-order harmonics appearing at the frequency multiplication position of the feature frequency. These higher-order harmonics may appear as additional peaks in the spectrum, revealing the severity and nature of the damage.

**Figure 6:**
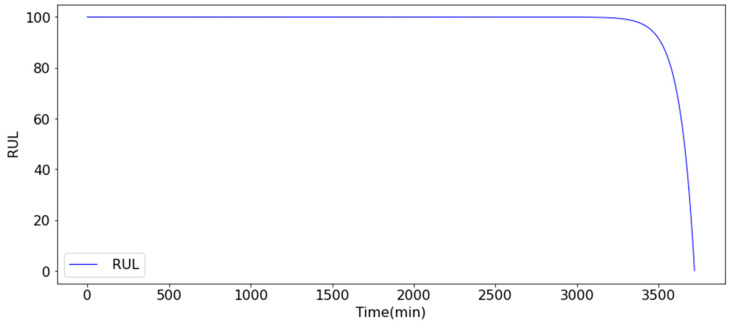
Health index (RUL) value.

The results demonstrate that spectrum analysis can effectively identify and differentiate bearing fault types. By inputting them into the Diagnosis and RUL Prediction Model, the feature values of bearings at each operational time point can be obtained. Subsequently, an exponential function (Equation (3)) is adopted to fit the feature values to obtain the corresponding RUL value for each operating time point (Figure 6). When the bearings are in a healthy state, the RUL remains at 100. The RUL value begins to decline after approximately 3400 min and may reach 0 after approximately 3650 min.

### 3.2. Practical Validation of the Ship Propeller Diagnosis and RUL Prediction System

**Figure 7:**
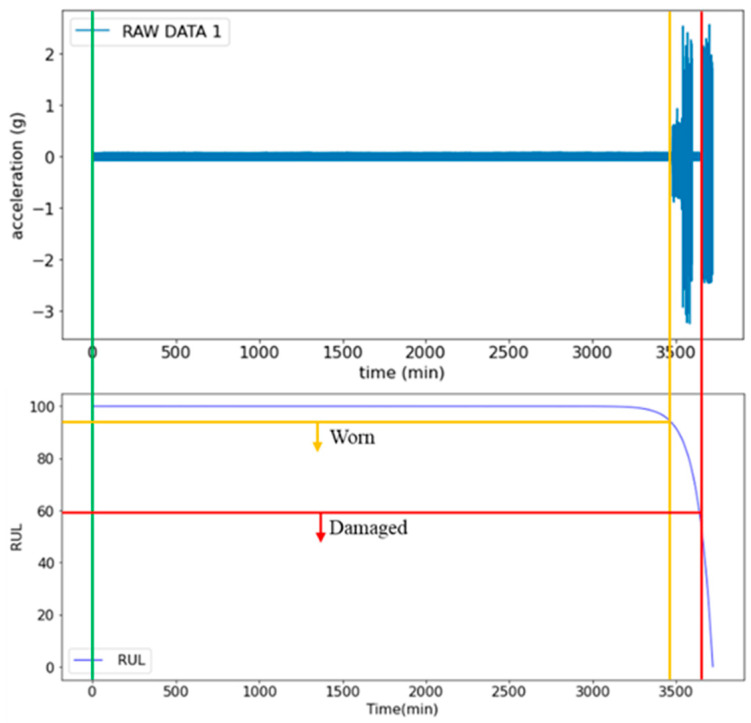
Signal changes of healthy, worn, and damaged bearings during rotation. The green line indicates the beginning of the data collection.

The ship propeller diagnosis and RUL prediction system involves inputting the vibration signals of propeller bearings into the Diagnosis and RUL Prediction Model to predict the RUL. The upper part of Figure 7 shows the changes in signals during the three stages of bearing, healthy, worn, and damaged, as well as the health index (RUL value). The health index value continuously decreases to a certain value, along with the wear of the bearing, revealing the changes of the bearing’s health status. Based on actual trials, as shown in the lower part of Figure 7, when the health index (RUL value) drops to 92, it indicates the onset of bearing wear. However, the degradation has not yet reached a critical level that significantly affects performance. This implies that while the bearing in the wear stage can still operate normally, it has entered a potential risk period for failure and should be closely monitored with timely maintenance. A health index below 40 suggests that the bearing has entered the damaged stage and requires immediate replacement or significant repairs. Otherwise, it may lead to severe equipment failure and unexpected shutdown. The judgment criteria obtained based on the results can be described as follows: when the health index drops below 97, the bearing is in a wear state, requiring routine inspections and preventive maintenance. A health index below 60 indicates that the bearing is in a damaged state, requiring immediate shutdown for replacement or repair. Based on the study findings, the threshold for triggering the early warning function should be adjusted from a health index of 40 to 60 to reduce the risk of failure.

**Figure 8:**
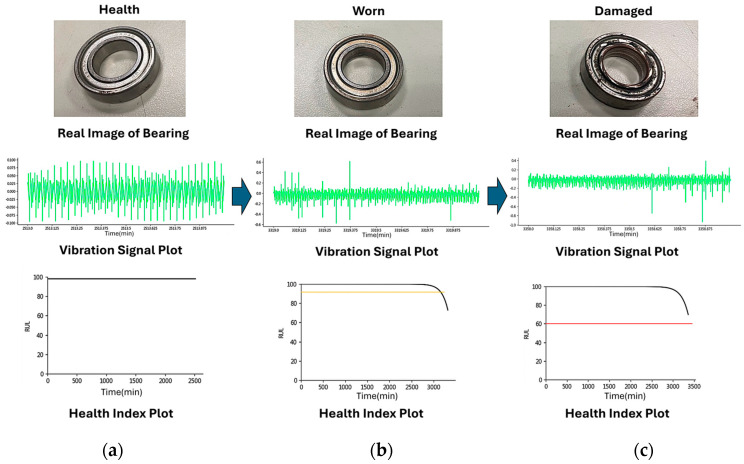
(**a**) Real images, (**b**) vibration signal plot, and (**c**) health index plot under different health states of ship propeller bearings during operations.

When applied in real-world diagnosis and RUL prediction, the proposed system can capture surface features, vibration signals, and health indexes of bearings at various stages (Figure 8). When the bearings are in a healthy state, the vibration signals fluctuate regularly and remain stable, while the health index stays at 100 until the bearings begin to wear, as shown in Figure 8a. When the vibration signals exhibit noticeable fluctuations and abnormalities, the progressively decreasing health index indicates that the bearings have transitioned from a healthy to a worn state; they require immediate maintenance and inspection to prevent further damage and equipment failure, as shown in Figure 8b. Finally, the vibration signals exhibit extreme fluctuations and abnormalities, with some vibration amplitudes significantly exceeding safe ranges and the health index values sharply declining, as shown in Figure 8c. In other words, the bearing has been damaged and requires immediate shutdown and repair. The experimental validation demonstrates that the ship propeller diagnosis and RUL prediction system can accurately detect bearing operational states and RUL. It can trigger early-warning functions, preventing unexpected equipment failure while facilitating maintenance operations. Thus, it addresses issues of insufficient ship maintenance capacity while enhancing operational efficiency.

The experimental results validate that the ship propeller diagnosis and RUL prediction system can accurately predict the RUL of bearings across differing operational scenarios, effectively estimating their health indexes (RUL values) under steady and abnormal vibration conditions. This is crucial for diagnosis and maintenance, as it can provide early warnings of equipment failure and reduce unexpected downtime while enhancing the safety and stability of equipment operations.

To ensure that the real-time prediction algorithm can accurately predict the remaining life under different operating conditions, load-altering experiments were conducted. In this experiment, the torque applied to the load screw was increased (from 2.8 Nm to 5.0 Nm) to enhance the stress endured by the test bearing.

**Figure 9:**
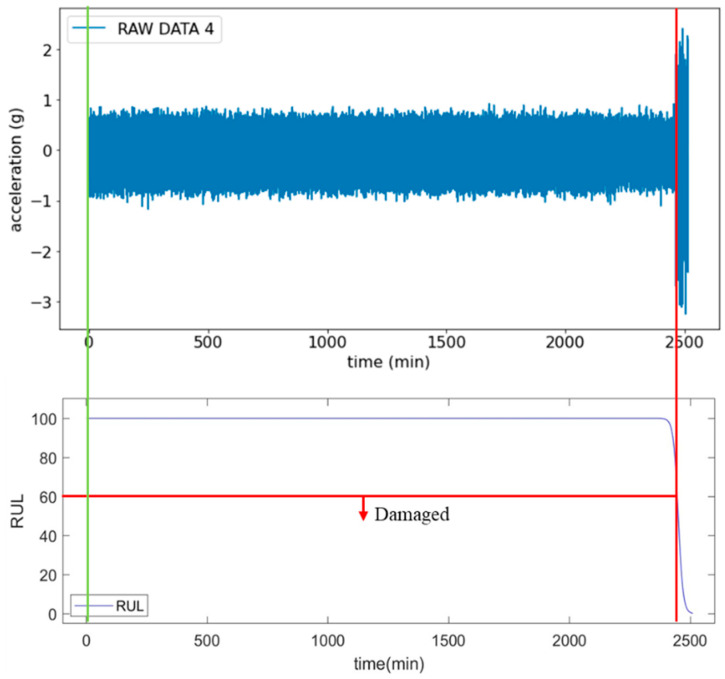
Signal plots and RUL prediction curves following load enhancement. The green line indicates the beginning of the data collection.

As illustrated in Figure 9, this adjustment results in a significant increase in the amplitude of the original vibration signals compared to the previous experiment. Starting from the 2398th minute, the predicted RUL displays a sharp decline. Moreover, by the 2408th minute, it decreases to below the critical threshold of 60, indicating that the bearing has entered a distinctly damaged phase. Unlike the previous experiment, the increase in load significantly expedites the wear and degradation process of the bearing, resulting in a substantial reduction in its use life and more obvious state changes. Furthermore, these abrupt changes are successfully captured by the LSTM model, enabling effective predictions of the bearing’s remaining use life. This is crucial for implementing predictive maintenance, as it allows for early warnings of equipment failures, thereby reducing unexpected downtime and significantly enhancing the safety and stability of equipment operation.

## 4. Conclusions

This study developed a ship propeller diagnosis and RUL prediction system and established a ship propeller sensing platform to capture bearing vibration signals of the propeller during operations. The input model, established using signal reshaping technology, was input into the trained Diagnosis and RUL Prediction Model to generate health index curves and predict the aging conditions and RUL of propeller bearings. Compared with RNN and SVR models, the MSE of the Diagnosis and RUL Prediction Model is 0.018, and the MAE is 0.039, exhibiting exceptional performance in prediction results and computational efficiency. Validation through ship propeller experiments shows that when the ship propeller operates normally, the model can analyze the bearing vibration signals in real-time to diagnose the healthy, worn, and damaged states, accurately judging the current RUL of the bearings. Thus, the ship propeller diagnosis and RUL prediction system can accurately predict the RUL of bearings under varying operational conditions, effectively estimating RUL values regardless of steady or abnormal signal fluctuations. It can enhance the operational efficiency of ships and allow for timely detection of potential faults, thereby improving operational reliability and extending the equipment’s predictable lifespan.

## Figures and Tables

**Table 1:** Comparative table of performance evaluation metrics.

Model Type	MSE ± Std Dev MSE	MAE ± Std Dev MAE
LSTM	0.018 ± 0.011	0.039 ± 0.022
RNN	0.236 ± 0.123	0.451 ± 0.123
Random forest	0.052 ± 0.021	0.203 ± 0.075
SVR	0.041 ± 0.002	0.197 ± 0.005

## Data Availability

The original contributions presented in this study are included in the article. Further inquiries can be directed to the corresponding authors.
